# Exploring the Interrelationships between Diabetes, Nutrition, Anxiety, and Depression: Implications for Treatment and Prevention Strategies

**DOI:** 10.3390/nu15194226

**Published:** 2023-09-30

**Authors:** Raedeh Basiri, Blessing Seidu, Mark Rudich

**Affiliations:** 1 Department of Nutrition and Food Studies, George Mason University, Fairfax, VA 22030, USA; 2 Institute for Biohealth Innovation, George Mason University, Fairfax, VA 22030, USA

**Keywords:** diabetes, mental health, anxiety, depression, supplementation, nutrition, blood glucose, cardiovascular health, cognitive decline, mental disorders, nutrition education

## Abstract

Across the world, diabetes, depression, and anxiety symptoms have gained widespread recognition as significant public health issues. Recent research has unveiled a mutually influential relationship between diabetes and these two mental health conditions, where each disorder impacts the course and outcomes of the others. The role of nutrition emerges as pivotal in preventing and treating depression, anxiety, and diabetes. A thorough literature review was undertaken to investigate the reciprocal effects between anxiety, depression, and diabetes, including their impact on the development and severity of each condition. Additionally, the effects of nutrition on the prevention and management of depression, anxiety, diabetes, and related complications in at-risk individuals were assessed. Our findings show that mental disorders, such as depression and anxiety, increase the risk of developing type 2 diabetes and are associated with poorer glycemic control, increased diabetes-related complications, and higher mortality rates. Conversely, diabetes is also linked with an increased risk of developing depression and anxiety. The biological, psychological, and social factors that contribute to the comorbidity between these two conditions are complex and multifaceted. Therefore, an integrated approach to the management of both conditions is critical for improving patient outcomes and reducing the overall burden of disease. Nutritional interventions should be utilized to reduce the risk of diabetes in patients with anxiety and depression as well as enhance mental health in patients with diabetes.

## 1. Introduction

According to the International Diabetes Federation, diabetes has been noted as one of the most devastating diseases in the 21st century, while many people remain undiagnosed [1]. Meanwhile, one in three adults has prediabetes [2]. The occurrence of diabetes is troubling as studies uncovered 463 million patients with diabetes in 2019, and the prevalence will potentially increase to 578 million by 2030 [3]. The incidence is expected to increase further, with estimates of more than 600 million people affected by 2040 [1]. As with most chronic diseases, many factors contribute to the occurrence and treatment of diabetes, including anxiety and depressive conditions.

Anxiety and depression have become widespread mental disorders identified by cognition, mood, and attitude variations that significantly impact an individual’s well-being and daily life [4,5]. Depression disorders have been estimated at approximately 17%, while the prevalence of anxiety disorders is about 29% [5,6,7]. Considerable evidence confirms that mental health disorders enhance appetite and cravings and decrease motivation for physical activity [8]. Furthermore, high sugar intake has been associated with depression and anxiety in numerous cross-sectional and observational studies [9,10,11,12]. Along the same lines, research studies have identified a link between the intake of refined carbohydrates and circulating inflammatory markers and their impact on mental health [13,14]. It has been reported that patients with mental disorders have a reduced intake of vegetables, fruits, whole grains, and fiber in their diet [15,16]. As a result, a low-quality diet consumed during episodes of depressive symptoms may elevate the risk of diabetes. Depression can also contribute to an increased risk of diabetes through biological mechanisms, particularly by impacting hormonal regulation associated with mood disorders [17]. Such processes include increased proinflammatory cytokines and abnormalities of the hypothalamic-pituitary-adrenal (HPA) axis [18]. Cortisol, one of the HPA axes hormones, is known to increase appetite and ramp-up motivation for the intake of calorically dense foods, which may increase blood glucose levels in at-risk populations [19,20].

While anxiety and depression may heighten the risk of developing diabetes or exacerbate its symptoms if the condition is present, it is essential to recognize that diabetes can likewise exert a reciprocal impact on these psychological states. Generally, individuals with poor glycemic status have a greater chance of developing anxiety and depression [2,17]. It has been shown that the prevalence rate of depression in individuals with prediabetes is three times and in patients with diabetes is two times higher than the general population [7]. Impaired glucose metabolism, management of symptoms, diminished response to treatment, and the fear of developing other complications could put patients with diabetes at higher risk of developing mental disorders and lower quality of life [21]. In adults with diabetes, 18% have been reported to require psychiatric support; however, it is also reported that 10% of these psychological conditions go undetected [22]. The duration of diabetes and glycemic control are significant determinants contributing to the emotional well-being challenges in adults diagnosed with diabetes [23]. Nutrition intervention offers a potential avenue for preventing or mitigating symptoms of depression, anxiety, and diabetes. The primary objective of this literature review was to examine the interplay between anxiety, depression and type 2 diabetes and investigate the role of nutrition in preventing or managing these interconnected conditions.

## 2. Materials and Methods

In this comprehensive literature review, we systematically selected studies based on specific criteria to investigate the intricate relationship between depression, anxiety, and diabetes and the potential influence of nutrition on these interconnected conditions. Various studies including observational studies, clinical trials, systematic reviews, and meta-analyses, were selected from reputable databases, such as PubMed, Google Scholar, and George Mason University Library. To encompass the most recent research developments, our search spanned from 2000 to 2023. A comprehensive set of keywords and phrases, including “diabetes”, “depression”, “anxiety”, “mental health”, “risk factors”, “symptoms”, “mechanisms”, and “nutrition,” were employed to maximize the effectiveness of the search. Only peer-reviewed articles were considered for inclusion to ensure study quality and reliability. Additionally, to enhance the rigor of our analysis, preference was given to articles employing validated measures of anxiety and depression such as the Beck Depression Inventory (BDI), Hamilton Depression Rating Scale and Hamilton Anxiety Rating Scale, Patient Health Questionnaire-9 (PHQ-9), and Generalized Anxiety Disorder-7 (GAD-7). In total, 120 articles were meticulously assessed, and 103 studies were included for their relevance to our research objectives, contributing to a comprehensive understanding of the complex interplay between mental health, diabetes, and the potential role of nutrition in the prevention and management of these conditions.

## 3. Results

Research has demonstrated a causal relationship between depression, anxiety, and diabetes. It has been shown that depression and anxiety are more prevalent among individuals with diabetes compared to healthy populations (26.3% vs. 11.2%, respectively) [24]. On the other hand, it has been suggested that individuals with established anxiety and depression are at increased risk of developing diabetes later in life [25]. A systematic review carried out in 2021 looked at the association of these psychiatric disorders with diabetes risk and found a relative risk of 1.60 for individuals with depression and an odds ratio of 1.47 for individuals with anxiety disorders [26].

### 3.1. Investigating the Effects of Diabetes on Depression Risk

Diabetes is associated with the onset and worsening of depression and overall depressive symptoms [27]. The occurrence of depression is two to three times higher in individuals with diabetes compared to individuals with normal blood glucose [28]. Approximately one in four adults with diabetes have depression, while only 25% to 50% of them are diagnosed and treated [29]. Treatment regimens for individuals with diabetes include the use of multiple medications, blood glucose monitoring, adherence to specific dietary guidelines, and attending regular medical check-ups [25]. These treatment approaches are aligned with the challenges faced by individuals dealing with diabetes or its associated complications. Patients with diabetes may experience a depressed mood, weight loss or gain, sleep disturbances, fatigue, stress, and decreased libido, which can have a dramatic effect on their mental health [30]. The relationship between living with diabetes and treating diabetes can give way to a foundation for the development of depression [30]. For patients with diabetes, even with high-quality treatment that could help to lessen the strain of diabetes, several factors still play a role in increasing the risk of depression; factors such as the psychological burden of being ill, unfavorable lifestyle, physician ignorance, or alterations in the activity of the hypothalamus–pituitary–adrenal (HPA) axis [31,32,33]. The HPA axis is an area of the brain that controls reactions to stress as well as regulates various body processes [33]. The interactions between the hypothalamus, pituitary gland, and adrenal glands can cause alterations to cortisol production, which could be an underlying mechanism of increased depression risk in people with diabetes [31,34].

The diagnosis of diabetes can be overwhelming for some people given the association it has with debilitating complications. In a study carried out by the European Depression in Diabetes (EDID) Research Consortium, patients diagnosed with diabetes were found to have higher incidences of depression suggesting that the knowledge of the diagnosis and the burden of treating the condition and its complications are associated with depressive symptoms [35]. This makes the case for doctors and healthcare providers to increase their role in the psychological aspects of their patient’s diagnoses. Another element contributing to depression is lifestyle factors such as sedentary behaviors and diets high in saturated fats and refined sugars, which may assist in priming or reinforcing the comorbidity of depression [35]. Additionally, it has been shown that poor glycemic control and greater glycemic variability are associated with reduced quality of life and negative moods [21]. This raises the possibility that there may be a mutually reinforcing phenomenon in which reduced self-care compliance increases blood glucose levels, which may contribute to depressive symptoms [35,36].

Controlling diabetes and depression together requires a multifaceted approach. Compared to those without depression, these individuals typically have higher glycemic levels, use health services more frequently, and have higher rates of complications [30]. A primary care study of 879 people with type 2 diabetes (T2DM) revealed that nonadherence to self-care management was causally related to hyperglycemia and diabetes complications [37]. Furthermore, individuals with diabetes and depression have been linked to a higher mortality rate [38].

### 3.2. Examining the Impact of Depression on Diabetes Risk

Not only does diabetes increase the risk of depression, but also individuals with depression might be at higher risk of diabetes. Depression is a major psychiatric illness that impairs the quality of life and puts enormous burdens on the healthcare system [39]. The behavioral, biological, and cognitive mechanisms associated with depression have been shown to increase the risk of diabetes development [40]. Health issues within these mechanisms seem to work in a continuous loop where they are in response to depression and a reason for the development of diabetes. Issues related to depression such as unhealthy eating habits, physical inactivity, or sleeping disturbances (behavioral) contribute to weight gain, increased activity in the HPA axis, and increased insulin resistance (biological), which can result in fatigue and a reduced ability to think and concentrate (cognitive) [26]. This may make individuals with depression less likely to participate in health-promoting behaviors such as healthy eating and exercise [41]. This loop could justify why there is a 60% increased risk of developing diabetes, specifically type 2, when also diagnosed with depression [42]. This is illustrated in a study conducted by Katon et al. (2009), which found that symptoms of depression were associated with fewer days of eating a healthy diet and exercising [43].

Further emphasizing the point of the behavioral connection between depression and the occurrence of diabetes was a study conducted by Golden et al. on ethnically diverse men and women, 45 to 84 years old [44]. This study looked at the relationship between depressive symptoms, associated lifestyle factors (e.g., physical inactivity, high-calorie diets), and the incidence rate of T2DM. The research findings revealed that individuals with elevated depressive symptoms had an incidence rate of 22.0 per 1000 person-years for developing type 2 diabetes, while those without such symptoms had an incidence rate of 16.6 per 1000 person-years. Additionally, the risk of incident T2DM was 1.10 times higher for each five-unit increment in the Center for Epidemiologic Studies Depression Scale (CES-D) score after adjustment for demographic factors and body mass index. The CES-D is a common self-reporting tool used to evaluate depression in the general population. Questions related to depression such as feelings toward mood, worthlessness, concentration, sleep, and lack of appetite are all assessed.

The biological mechanisms associated with depression are also linked to an increased risk of diabetes. An illustration of this is the association between depression and heightened activity of the hypothalamic–pituitary–adrenal (HPA) axis and sympathetic nervous system (SNS), as well as increased production of proinflammatory cytokines [41]. Elevated activity of the HPA axis and SNS leads to heightened production of the stress hormone cortisol, contributing to increased glucose production and reduced insulin sensitivity. Prolonged hypercortisolemia further raises the risk of developing metabolic syndrome, characterized by central adiposity, excessive accumulation of abdominal fat, and insulin resistance [45]. Cortisol’s rise also increases one’s risk of developing diabetes [7,41]. Moreover, chronic stress can trigger immune dysfunction either directly or through the activation of the HPA axis or SNS. This leads to an upsurge in the production of inflammatory cytokines that disrupt the normal functioning of pancreatic β-cells, promote insulin resistance, and consequently contribute to the onset of diabetes [7]. Studies have shown a significant association between elevated C-reactive protein (CRP), a pro-inflammatory cytokine, and diabetes risk [7,46] as well as related complications [47]. Overall, the role of behavior, biology, and cognition in the development of diabetes highlights the bidirectional relationship between diabetes and depression.

### 3.3. The Effects of Diabetes on Anxiety Risk

Growing evidence has established a synergy between diabetes and anxiety [48]. Individuals living with diabetes are at increased risk of developing anxiety because of concerns about dietary restrictions, taking several medication and insulin injections, constant checking of blood glucose, and lack of support from family and medical practitioners [49]. As such, incorporating psychological care in the management of diabetes could aid in reducing anxiety as emphasized by the International Federation of Diabetes [50,51]. In patients with diabetes, comorbid anxiety syndromes are correlated with heightened blood glucose levels, high body-mass index (BMI), and greater disability [52,53,54]. Cure of anxiety is linked to enhanced glycemic control [22], especially among people affected with acute anxiety [23]. Unfortunately, many people with anxiety are undiagnosed, and hence untreated [55]. Undiagnosed anxiety among people with diabetes is concerning because it delays the onset of therapy for these coexisting illnesses and causes patients to become frustrated, which leads to ineffective therapeutic results [56].

The prevalence of anxiety in diabetes seems to be increasing rapidly. In one study, individuals with diabetes had a 20.6% prevalence of anxiety in 2000, and by 2004 the prevalence had risen to 42.2% [57]. In the same study, it was identified that people aged ≥45 were linked to a higher incidence, while females were associated with a more significant occurrence with age ≥45 [57]. In another study, compared to women without diabetes, women with diabetes had a greater lifetime incidence of anxiety disorders, with 50% having had an anxiety disorder at some point in their lives [31]. Moreover, both males and females showed a correlation between T2DM, obesity, and anxiety, with more than one in three women and one in five men showing signs of anxiety in research findings [58].

Most people with diabetes who are hospitalized have been observed to experience moderate to severe anxiety, sadness, or both throughout their stay [59]. Research studies have demonstrated that anxiety can influence medication adherence in patients with diabetes through its impact on self-efficacy expectations, which refers to an individual’s belief in their ability to successfully carry out specific tasks or activities [60]. This can lead to worsened glucose regulation and greater difficulties in diabetes management [28]. The coexistence of diabetes and anxiety is associated with an elevated risk of developing comorbidities, increased healthcare costs, and early morbidity and mortality [61,62].

### 3.4. The Effects of Anxiety on Diabetes Risk

Anxiety is a mental health disorder characterized by behavioral syndrome, fear, and feeling a sense of panic, with distinct subtypes of anxiety traits [63]. Research studies show that generalized anxiety disorder (GAD) and panic disorder are the most common mental health conditions [64]. A study examined the use of depression and anxiety screenings and each of their respective components as concurrent predictors of the onset of diabetes [37]. It was noted that 24.9% of the participants with anxiety at baseline developed diabetes over 10 years [37]. Screening for both diabetes and anxiety is highly recommended for patients, as anxiety not only serves as a risk factor for the development of diabetes but can also coexist as a comorbidity [37].

In a recent study to investigate the occurrence of diabetes among individuals with anxiety, it was discovered that diabetes was more common among patients with anxiety as compared to the general population (11.89% vs. 5.92%, odds ratio, 1.23; 95% confidence interval, 1.17–1.28) [65]. The average annual occurrence of diabetes in patients with an anxiety disorder between 2006–2010 was revealed in another study demonstrating a higher prevalence of diabetes among people with anxiety disorders as contrasted with the general population (2.25% vs. 1.11%, risk ratio 1.34; 95% confidence interval, 1.28–1.41) [65].

Delving deeper into the link between anxiety and diabetes, research studies have identified gender as a contributing factor in diabetes prevalence [66]. Women are observed to be more at risk of developing diabetes than men, as women tend to have higher levels of anxiety disorders than men and men handle anxiety-related conditions differently than women [67,68]. Other discoveries have also suggested that the higher ratio of women compared to men in diabetes occurrence is due to a higher percentage of women with mental health issues such as depression and anxiety [67]. In a prospective study investigating the association between mental health and diabetes, individuals with reported symptoms of anxiety and depression at baseline had a higher risk of the occurrence of T2DM at a ten-year follow-up [69]. Dealing with anxiety influences eating habits [70] and eventually results in increased adiposity and risk of developing diabetes [71]. Both anxiety and diabetes-related distress are linked to suboptimal glycemic control, decreased adherence to diabetes self-care routines, and an increased likelihood of developing diabetes complications [72]. A summary of the results of related studies on the interrelationship between diabetes, depression, and anxiety have been reported in Table 1.

### 3.5. Exploring the Role of Nutrition in Preventing and Managing Diabetes, Depression, and Anxiety

Poor nutritional status and unhealthy lifestyle habits have been widely acknowledged as established risk factors contributing to the pathogenesis of diabetes [78,79,80]. For instance, a study of 200,000 participants found that a healthy diet, characterized by a high intake of whole grains, vegetables, fruits, and nuts, and a low intake of red and processed meats, was correlated with a decreased risk of T2DM [81]. Another study found that a diet high in sugar-sweetened beverages and processed foods was associated with an increased risk of diabetes [82]. Moreover, nutrition plays a crucial role in the management of diabetes and related complications [47,83,84,85,86,87]. A systematic review found that a Mediterranean diet, which is high in vegetables, whole grains, fruits, lean protein, and healthy fats, improved glycemic control and reduced cardiovascular risk factors in patients with T2DM [88]. An alysis of data from three European cohorts and one Canadian cohort including 78,851 participants showed that higher protein intake was correlated with a lower risk of diabetes [89]. More specifically, plant protein intake was significantly and negatively related to the development of diabetes [90]. Other studies found that a low-carbohydrate diet was effective in improving glycemic control and reducing the need for diabetes medication in patients with T2DM [91,92].

Similarly, consuming high-quality diets is associated with improved mental health [93]. In a cross-sectional analysis involving a cohort of 3172 adults between the ages of 18 and 55, it was observed that adhering to the Mediterranean diet exhibited a significant correlation with a decreased likelihood of experiencing psychological disorders such as depression, anxiety, and psychological distress [94]. Another study found that a high glycemic index diet is a risk factor for depression [9]. In contrast, it has been shown that a high-fiber diet improves glucose homeostasis, serum lipid profiles, inflammatory chemokines, and depression and anxiety symptoms in patients with T2DM [95]. Evaluating the effects of different sources of protein on the mental well-being of women showed that women who consumed more animal protein had a higher chance of showing symptoms of depression (OR: 2.63; 95% CI: 1.45, 4.71; *p*  =  0.001), stress (OR: 3.66; 95% CI: 2.06, 6.50; *p*  <  0.001), and anxiety (OR: 1.83; 95% CI: 1.04, 3.22; *p*  =  0.03), while no significant association was found between plant protein and these mental disorders [96]. Polyunsaturated fats have also been found to be effective in reducing symptoms of depression and anxiety. For example, a randomized controlled trial found that omega-3 fatty acid supplementation significantly reduced symptoms of depression in patients with diabetes who showed mild to moderate depressive symptoms, independent of metabolic factors and disease duration [97].

Scientific evidence reveals a significant association between the consumption of ultra-processed foods and the occurrence of depression and other mental disorders [98,99]. A meta-analysis of prospective studies supports this link, indicating that higher intake of ultra-processed foods is associated with an increased risk of subsequent depression (hazard ratio: 1.22, 95% CI 1.16 to 1.28) [100]. These findings emphasize the potential detrimental impact of consuming ultra-processed foods on psychological outcomes, underscoring the importance of considering dietary choices in promoting mental well-being.

**Figure 1:**
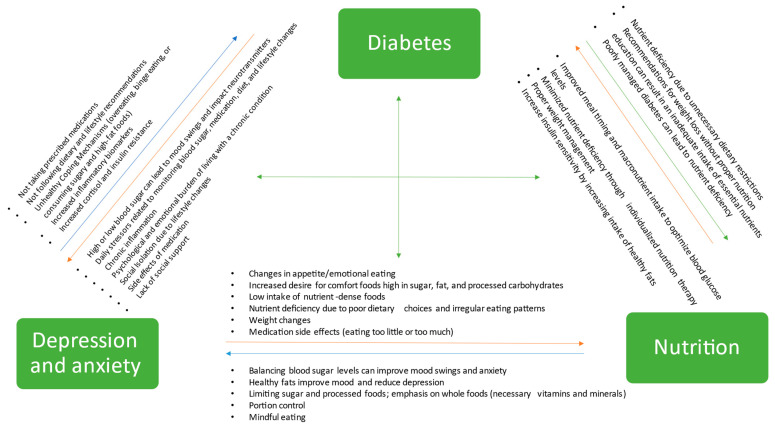
Interplay of diabetes, depression, anxiety, and nutrition: a comprehensive overview.

Overall, there is growing evidence to support the link between nutrition, diabetes, and depression and anxiety. A healthy and balanced diet, characterized by a high intake of whole grains, fruits, vegetables, nuts, lean protein, and healthy fats, reduce the risk of diabetes, improve glycemic control in patients with diabetes, prevent and manage related complications, and improve mental health. Furthermore, certain eating behaviors, such as a high intake of fiber, a low glycemic diet, high intake of unsaturated fats including omega-3 fatty acids and plant-based proteins, may play a crucial role in reducing the risk of developing diabetes and anxiety and depression, and can alleviate symptoms of these diseases if they exist. Aside from the effects of eating patterns, certain nutrients play an important role in preventing or alleviating symptoms of diabetes, anxiety, and depression. Deficiencies in certain essential nutrients, including omega-3 fatty acids, vitamin D, B vitamins, zinc, chromium, magnesium, and selenium, have been associated with the development of both diabetes and mental health disorders [101]. In the realm of mental health treatment in patients with diabetes, there is a noticeable scarcity of randomized trials that assess the effectiveness of dietary interventions in these conditions. The Mediterranean diet has proven effective in mitigating symptoms of depression, anxiety, and diabetes when studied separately. However, its effects have not yet been investigated in individuals who simultaneously suffer from both conditions. A notable example of the effects of the Mediterranean diet on the symptoms of anxiety and depression involves a 12-week Mediterranean diet intervention study, which yielded substantial improvements in mood and reduced anxiety among adults diagnosed with major depression [102]. Another randomized controlled trial has reaffirmed the positive impact of a Mediterranean-style diet on mental health, particularly in the context of depression [103]. The Mediterranean diet has also demonstrated its advantages in mitigating several cardiovascular risk factors, with a particular focus on its positive impact on diabetes [88]. These findings highlight the importance of a healthy diet in promoting overall health and well-being, especially for individuals with diabetes and or anxiety and depression. Figure 1 shows the interplay of diabetes, depression, anxiety, and nutrition.

## 4. Discussion

Studies have shown that individuals with mental health disorders, such as depression and anxiety, are at a heightened risk of developing diabetes due to biological, behavioral, and cognitive factors. Conversely, receiving a diabetes diagnosis can lead to significant lifestyle adjustments and emotional distress, potentially exacerbating mental health issues and blood glucose control difficulties. Persistent anxiety about managing blood glucose levels can activate the HPA axis, increasing blood glucose levels and the risk of related complications. Furthermore, individuals with anxiety or depression may experience appetite changes, potentially worsening diabetes management and complications risk. Comprehensive healthcare should address symptoms of depression and anxiety in individuals with diabetes, as well as screen those with these psychological disorders for diabetes symptoms. Lifestyle modifications, therapy, and medications, when necessary, should be considered to address risk factors for both mental health conditions and diabetes. Nutrition plays a pivotal role in mitigating symptoms of these conditions by stabilizing blood glucose levels, providing essential nutrients, and supporting weight management; therefore, dietary recommendations should be embraced to support individuals in maintaining optimal blood glucose control, reducing diabetes complications, enhancing emotional resilience, and improving the overall quality of life in these populations.

Medications prescribed for these conditions can impact nutritional status, with certain medications affecting appetite, nutrient absorption, or dietary restrictions. The complexity of medication interactions and their collective nutritional impact becomes more pronounced in individuals with comorbid conditions. Tailored medication management and dietary recommendations aligned with an individual’s specific medical and nutritional needs are pivotal for improving the well-being of individuals managing diabetes alongside depression and anxiety. It is important to note that this study’s limitation lies in the unavailability of participants’ medication information, preventing an assessment of medication influences on the complex interplay between nutrition, diabetes, anxiety, and depression.

A comprehensive understanding of the intricate interactions between diabetes, mental health, and nutrition is vital for holistic patient care. While cross-sectional studies highlight positive associations among diabetes, anxiety, and depression, longitudinal research reveals potential bidirectional causal relationships. It is imperative to recommend longitudinal studies to explore each condition’s influence on the others and assess the impact of interventions targeting one condition on the rest. Personalized nutrition interventions should also be incorporated into future research to evaluate their effectiveness when managing comorbid diabetes, depression, and anxiety.

## 5. Conclusions

Our findings have shed light on the bidirectional relationship between diabetes, depression, and anxiety, highlighting the importance of early detection and comprehensive management to reduce related complications. Nutrition emerges as a powerful tool in this context, offering a pathway to better diabetes control and improved mental health. Further research is needed to elucidate the specific mechanisms by which nutrition impacts these interconnected conditions, paving the way for more tailored interventions and enhanced well-being.

## Figures and Tables

**Table 1:** The interrelationship between diabetes and mental disorders.

Author	Study Design	Population	Results
Rajput et al., 2016 [25]	Cross-sectional, case–control study design.	Diabetes, *n* = 410 Healthy control, *n* = 410	There were twice as many cases of depression and anxiety in those with T2DM compared with healthy controls (26.3% vs. 11.2%).
Tovilla-Zarete et al., 2012 [73]	Cross-sectional, multi-center study	Diabetes, *n* = 820	48.27% were positive for depression while 55.10% showed symptoms of anxiety. Occupation and complications were associated with anxiety while glucose level and complications were correlated with depression.
Collins et al., 2009 [74]	Cross-sectional study	Diabetes, *n* = 1456	Patients with diabetes had significant levels of anxiety (32.0%) and depressive symptoms (22.4%). Poor glycaemic control and female gender were risk factors for higher anxiety scores. Older age and a higher socioeconomic status demonstrated a protective effect, resulting in lower scores of anxiety and depression.
Shaban et al., 2006 [75]	Cohort	Diabetes, *n* = 273	Compared to men, women reported considerably greater mean anxiety levels. HbA1c was positively associated with anxiety and depression.
Campayo A. et al., 2010 [40]	longitudinal design	Subjects with depression, *n* = 379	Severe, moderate, and untreated depression are all linked to an increased risk of developing type 2 diabetes. Although persistent depression had a greater risk than the rest.
Golden S.H. et al., 2008 [44]	Longitudinal cohort study	Analysis 1. participants without type 2 diabetes at baseline with and without depressive symptoms, *n* = 5201 Analysis 2. participants without depression at baseline with and without type 2 diabetes, *n* = 4847	The incidence of diabetes was correlated with baseline depressed symptoms. Impaired fasting glucose and untreated type 2 diabetes were negatively correlated with incident depressive symptoms, while treated type 2 diabetes exhibited a positive association.
Iversen et al., 2015 [76]	Cohort	Depression, *n* = 36,031	Depression was positively associated with diabetes.
Chien I. C & Lin C. H, 2016 [65]	Prospective cohort	subjects had primary and secondary diagnoses of anxiety disorder, *n* = 766,427	The prevalence of diabetes among individuals with anxiety disorders was greater than that of the general population.
Smith et al., 2018 [77]	Meta-analysis	anxiety, 14 studies (*n*= 1,760,800)	Substantial positive correlation was shown between baseline anxiety and the incidence of diabetes
Khambaty T., 2017 [37]	Cohort	anxiety, *n* = 2156	Out of 2156 patients, 558 developed diabetes over a 10-year period.
Engum A., 2007 [69]	Prospective population-based study	depression and anxiety, *n* = 8311	Individuals with reported baseline symptoms of depression and anxiety were more likely to develop diabetes ten years later.
Meurs et al., 2016 [24]	Cohort study	Depression *n* = 3002 Anxiety *n* = 9018 Diabetes *n* = 1781 Undiagnosed diabetes *n* = 786	Diabetes was independently linked to depression in both identified and undiagnosed cases. Diabetes diagnosis was independently linked to anxiety but this association was not observed for undiagnosed cases.

## Data Availability

Not applicable.
